# Thyroid hormones profile among obese pregnant Sudanese women

**Published:** 2020-07-08

**Authors:** Wisal Abbas, Ishag Adam, Duria A. Rayis, Nada G. Hassan, Mohamed F. Lutfi

**Affiliations:** ^1^Department of Physiology, Faculty of Medicine, Al-Neelain University, Khartoum, Sudan; ^2^Department of Obstetrics and Gynecology, Unaizah College of Medicine and Medical Sciences, Qassim University, Unaizah, Kingdom of Saudi Arabia; ^3^Department of Physiology, College of Medicine, Qassim University, Kingdom of Saudi Arabia; ^4^Department of Physiology, Nile College of Medicine, Khartoum, Sudan

**Keywords:** Body mass index, Obesity, Pregnancy, Sudan, Thyroid hormones

## Abstract

**Background:**

Previous studies evaluating thyroid function among obese pregnant women failed to demonstrate a consistent pattern of thyroid hormones profile, probably due to the variations in biological/environmental determinants of thyroid function in different countries.

**Aim:**

The aim of the study was to evaluate thyroid hormones profile in Sudanese pregnant women with varying degrees of obesity.

**Patients and Methods:**

Obstetric/sociodemographic characteristics were gathered from 178 singleton pregnant Sudanese women using questionnaires. Weight and height were measured; body mass index (BMI) was calculated and categorized into four groups: Underweight (BMI <18.5 kg/m^2^), normal weight (18.5-24.9 kg/m^2^), overweight (25.0-29.9 kg/m^2^), and obese (≥30 kg/m^2^). Free triiodothyronine (FT3), free thyroxin (FT4), and thyroid-stimulating hormone (TSH) were measured.

**Results:**

Of the 178 enrolled women, 9 (5.1%), 52 (29.2%), 73 (41.0%), and 44 (24.7%) were underweight, normal BMI, overweight, and obese, respectively. FT3 level was significantly higher in obese women compared with normal BMI (*P*=0.004) as well as overweight women (*P*=0.015). Higher FT3 levels were significantly associated with obesity (odds ratio [OR]=9.5, 95% confidence interval [CI] =3.1-29.0, *P*<0.001). Lower levels of FT4 were significantly associated with overweight (OR=0.06, 95% CI=0.007-0.58, *P*=0.015) and obesity (OR=0.048, 95% CI=0.004-0.5, *P*=0.018). Based on linear regression analysis, BMI was positively associated with FT3 (4.7 pmol/l, *P*<0.001) and negatively associated with FT4 (−8.26 pmol/l, *P*=0.001).

**Conclusions:**

BMI correlates with FT3 differently compared to FT4. Pregnant women with higher BMI are likely to have higher levels of FT3, but lower FT4. In contrast, TSH levels were comparable in different BMI groups.

**Relevance for Patients:**

Increased iodothyronine 5´deiodinase (5´D) activity associated with obesity may give an explanation for thyroid profile in those with higher BMI. High 5´ activity increases FT3 at the expense of FT4. Alternatively, high FT3 and low FT4 are expected to feedback differently on TSH, which explains the loss of positive correlation between BMI and TSH.

## 1. Introduction

Maternal obesity is a public health problem worldwide. About one-third of pregnant women in the US are overweight and another third are obese [1,2]. In China, the prevalence of overweight women is comparable to the UK and the US, though the prevalence of obese women is as low as 5.8% [3]. In Africa, the prevalence of maternal obesity ranged from 6.5% to 50.7% [4], being higher in Sudan compared to other sub-Saharan countries [4-7]. Available data demonstrated prevalence rates as high as 35.6% and 19.4% of overweight and obesity among pregnant Sudanese women at term, respectively [7].

Although the undesirable effects of obesity on the obstetric outcome are well studied [8-10], contribution of thyroid dysfunction in the pathophysiology of maternal obesity remained ill-defined during pregnancy and deserved further research. Thyroid hormones are known to regulate appetite, thermogenesis, basal metabolic rate, and consequently body weight [11,12]. Studies evaluating thyroid hormones profile among pregnant women with varying degrees of obesity showed non-reproducible results and failed to follow a consistent pattern [1,2,13-19]. Variations of maternal characteristics, accuracy of the methods used for thyroid hormonal assays, metabolism of thyroid hormones in obese subjects, and interaction between thyroid hormones, and other regulators of body weight may explain these non-reproducible results [20-25].

Thyroid hormones profile demonstrates circadian rhythm [26] and seasonal variation [27] and is affected by ambient temperature [28] as well as other environmental/genetic factors [29,30]. Influences of these biological/environmental factors on thyroid function give another explanation for the possible differences in thyroid hormones profile of obese pregnant women in different countries. The present study aims to evaluate thyroid profile, namely, free triiodothyronine (FT3), free thyroxin (FT4), and thyroid-stimulating hormone (TSH) in obese pregnant Sudanese women. We think that the results of the present study will add to the literature regarding the role thyroid hormones in the pathophysiology of obesity. This is especially true if we consider the unique tropical environment of Sudan and the relatively high prevalence rates of overweight and obesity among pregnant Sudanese women compared with other African countries [7]. The findings of the current study will add to our recent findings on the trimester-specific reference ranges for TSH, FT4, and FT3 in pregnant Sudanese pregnant women [31].

## 2. Patients and Methods

A cross-sectional study was conducted at Saad Abu-Alela Maternity Hospital, Khartoum, Sudan, during the period of January to April 2015. Saad Abu-Alela Hospital is a tertiary semi-private hospital governed by the Faculty of Medicine, University of Khartoum. The study was performed according to the recommendations of Research Board at the Department of Obstetrics and Gynecology, Faculty of Medicine, University of Khartoum, Sudan, with written informed consent from all subjects. The protocol was approved by the ethics review committee, Faculty of Medicine, University of Khartoum, Sudan.

After signing informed consent, eligible women who attended their first antenatal care clinics in the first trimester (<14 weeks) of gestational age were enrolled in the study. Inclusion criteria were: Singleton pregnancy and willingness to participate in the study. Women with a twin pregnancy and those with known thyroid disease or taking thyroid medication were excluded from the study. A trained medical officer used a pre-tested questionnaire to gather data from each pregnant woman on her age, parity, educational level (illiterate, primary school education or secondary school, and higher education), occupation (housewife or working mother), gestational age calculated in weeks, and medical diseases such as diabetes and hypertension. Weight and height were determined, and body mass index (BMI) was calculated and expressed as a weight (kg)/height (m)^2^.

BMI was categorized into four groups: Underweight (<18.5 kg/m^2^), normal weight (18.5-24.9 kg/m^2^), overweight (25.0-29.9 kg/m^2^), and obese (≥30 kg/m^2^) following classification of the World Health Organization, which addresses obesity during pregnancy as well as the general population [32].

For measurements of maternal serum thyroid profile (FT3, FT4, and TSH) levels, a volume of 5 ml of venous blood was taken from each pregnant women into plain serum bottles, allowed to clot, centrifuged to separate the serum, and stored at −20°C until measurement of serum thyroid hormones levels using the immunoassay analyzer AIA 360 (Tosoh Bioscience, San Francisco, CA, USA), guided by the manufacturer’s instructions as described previously [33]. All samples were performed in duplicate and the mean of the two was used as the final one. The reference intervals for TSH, FT3, and FT4 for the laboratory were: TSH, 0.38-4.3 IU/ml; FT3, 3.23-5.13 pmol/l; and FT4, 10.55-20.9 pmol, The details of normal value for TSH, FT3, and F T4 among pregnant Sudanese women have been recently published [31].

A total sample size of 178 participants was calculated using the previous incidence of obesity (19.4%) among pregnant women in Sudan [7]. A formula was used to calculate the difference in the mean of the proposed variable (FT3, FT4, and TSH in the obese and normal-weight women) that would provide 80% power to detect a 5% difference at α=0.05, with an assumption that complete data might not be available for 10% of participants.

SPSS for Windows (version 20.0) was used for data analyses. Kolmogorov-Smirnov test was used for testing the normality of the data. One-way analysis of variance (ANOVA) (with post hoc Bonferroni tests) and Chi-square tests were used to compare the normally distributed continuous variables and proportions between BMI groups, respectively. Kruskal-Wallis H test (Mann-Whitney U for two variables) was used to test non-parametric continuous data (FT3, FT4, and TSH). Multinomial logistic regression was conducted, where BMI group was the dependent variable and sociodemographic parameters (age, parity, education, job, and residence), gestational age, hemoglobin levels, TSH, FT3, and FT4 were the independent variables. Odds ratio (OR) and 95% confidence interval (CI) were calculated using pregnant women with normal weight as a control group. Linear regression models were conducted, where the log of TSH, FT3, and FT4 were the dependent variables, and age, parity, education, job, residence, gestational age, BMI, hemoglobin levels were the independent variables. *P*<0.05 was considered statistically significant at a two-sided test.

## 3. Results

General characteristics of the 178 enrolled women are shown in Table 1. Around two-fifth (n=80, 44.9%) of the women were primiparae. The mean (SD) of the age, parity, and gestational age was 27.5 (5.5) years, 1.0 (1.2), and 10.6 (3.4) weeks, respectively.

**Table 1:** General characteristics of Sudanese women in the current study

Variable	*n*=178
The mean (SD) of	
Age, years	27.5 (5.5)
Parity	1.0 (1.2)
Gestational age, weeks	10.6(3.4)
Body mass index, kg/m^2^	27.3(6.5)
Hemoglobin, g/dl	10.9(0.9)
Number (%) of	
Rural residence	47(26.4)
Education level ≤secondary level	19 (10.7)
Non-employees (housewives)	141 (79.2)

Of the 178 enrolled women, 9 (5.1%), 52 (29.2%), 73 (41.0%), and 44 (24.7%) were underweight, normal BMI, overweight, and obese, respectively (Table 2).

**Table 2:** Comparison of sociodemographic, medical, and obstetric characteristics between BMI groups among pregnant Sudanese women.

Variables	Underweight *n* (9)	Normal *n* (52)	Overweight * n* (73)	Obese * n* (44)	*P*
The mean (SD)					
Age, years	24.8 (5.2)	26.6 (6.4)	27.3 (5.2)	29.4 (4.8)	0.037
Parity	0.2 (0.4)	0.9 (1.0)	1.0 (1.3)	1.2 (1.1)	0.1421
Gestational age, weeks	30.6 (2.5)	32.1 (3.7)	32.3 (3.7)	32.0 (2.9)	0.610
Body mass index, kg/m^2^	16.7 (0.7)	22.6 (1.6)	27.1 (1.4)	35.3 (7.1)	<0.001
Hemoglobin, g/dl	10.3 (0.9)	10.9 (0.9)	10.9 (0.8)	10.9 (1.0)	0.343
Number (%)					
Rural residence	2 (22.2)	21 (40.4)	13 (18.1)	11 (25.0)	0.048
Education level ≤secondary level	0 (0)	6 (11.5)	6 (8.2)	7 (15.9)	0.417
Housewives	8 (88.9)	42 (80.8)	56 (76.7)	35 (79.5)	0.833
Median (interquartile range)					
TSH, IU/ml	1.899 (1.280-2.088)	1.813 (1.410-2.367)	1.803 (1.218-0.520)	1.605 (1.089-0.962)	0.304
FT3, pmol/l	1.700 (1.565-1.920)	1.830 (1.467-2.197)	1.950 (1.500-2.215)	2.090 (1.800-2.382)	0.001
FT4, pmol/l	1.100 (0.955-1.445)	1.070 (0.855-1.180)	0.990 (0.815-1.130)	0.985 (0.892-1.097)	0.497

There was no significant difference in residence, education, occupation, parity, and gestational age; however, age was significantly higher in obese than normal BMI group (Table 2).

**Figure 1:**
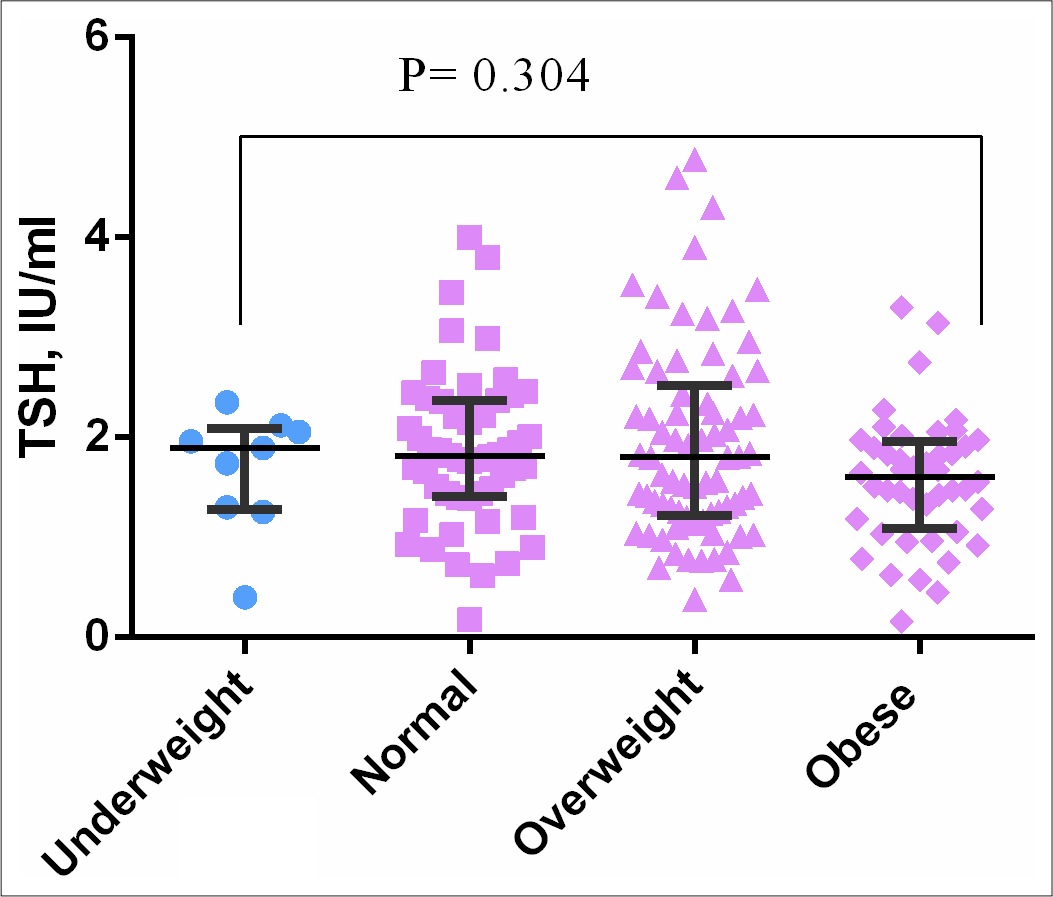
Distribution of thyroid-stimulating hormone among pregnant woman with different body mass index.

**Figure 2:**
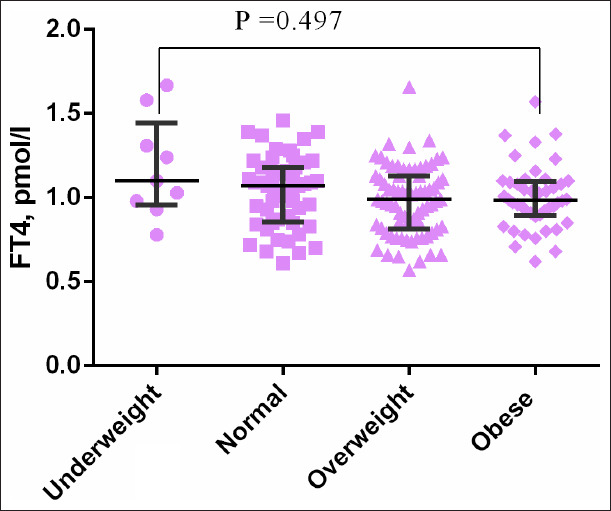
Distribution of free tetraiodothyronine among pregnant woman with different body mass index.

**Figure 3:**
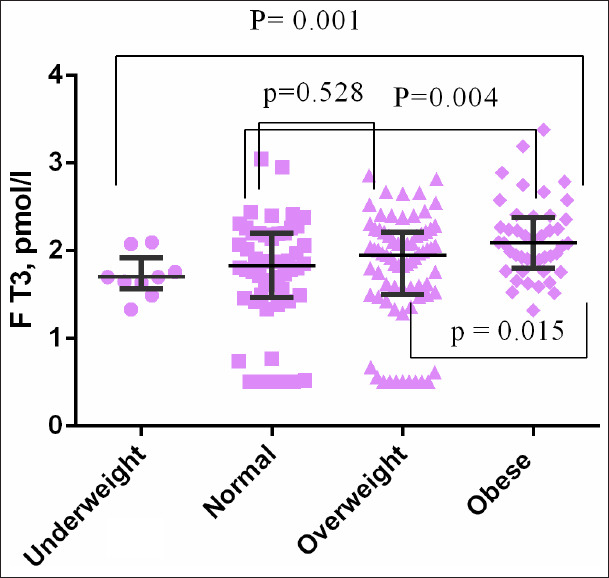
Distribution of free tetraiodothyronine among pregnant woman with different body mass index.

There was no significant difference in the level of TSH (Figure 1) and FT4 (Figure 2) between BMI groups. FT3 level was significantly higher in obese women compared with normal BMI women (*P*=0.004) and it was significantly higher in obese compared with overweight women (*P*=0.015) (Figure 3). However, there was no significant difference in FT3 level between overweight women and normal BMI women (*P*=0.528) (Table 2, Figure 1).

Compared to normal BMI women, higher FT3 levels were significantly associated with obesity (OR=9.5, 95% CI=3.1-29.0, *P*<0.001). In contrast, lower levels of FT4 were significantly associated with overweight (OR=0.06, 95% CI=0.007-0.58, *P*=0.015) and obesity (OR=0.048, 95% CI=0.004-0.5, *P*=0.018) (Table 3).

**Table 3:** Multinomial analyses of the factors associated with overweight and obese groups where normal weight was the reference

Variables	Overweight (*n*=73)		Obese (*n*=44)	
	
	OR (95% CI)	*P*	OR (95% CI)	*P*
Age, years	0.9 (0.9-1.0)	0.904	1.1 (1.02-1.2)	0.019
Parity	1.1 (0.7-1.6)	0.636	1.0 (0.6-1.5)	0.972
Gestational age, weeks	1.0 (0.9-1.1)	0.456	1.0 (0.9-1.2)	0.265
Hemoglobin, g/dl	1.0 (0.6-1.5)	0.829	1.1 (0.6-1.8)	0.587
Rural residence	4.0 (1.6-10.2)	0.002	2.8 (0.9-8.5)	0.057
Education level ≤secondary level	0.7 (0.1-2.5)	0.591	1.2 (0.3-5.3)	0.743
Housewives	0.9 (0.3-2.7)	0.945	2.4 (0.6-8.8)	0.178
TSH, IU/ml	1.0 (0.6-1.6)	0.801	0.5 (0.2-1.0)	0.054
FT3, pmol/l	2.1 (0.8-5.2)	0.098	9.5 (3.1-29.0)	<0.001
FT4, pmol/l	0.06 (0.01-0.6)	0.015	0.05 (0.004-0.5)	0.018

FT3: Free triiodothyronine, FT4: Free tetraiodothyronine, TSH: Thyroid-stimulating hormone

Using linear regression, TSH was not significantly associated with BMI. Alternatively, BMI was positively associated with FT3 (4.716 pmol/l, *P*<0.001) and negatively associated with FT4 (−8.26 pmol/l, *P*=0.001) (Table 4).

**Table 4:** Linear regression analysis of the factors associated with body mass index

Variables	Coefficient	Standard error	*P*
Age, years	0.151	0.106	0.156
Parity	0.523	0.473	0.27
Gestational age, weeks	0.253	0.149	0.091
Hemoglobin, g/dl	0.311	0.501	0.535
Rural residence	−0.629	1.09	0.565
Education level ≤secondary level	0.116	1.559	0.941
Housewives	−0.627	1.279	0.625
TSH, IU/ml	−0.493	0.568	0.387
FT3, pmol/l	4.716	0.975	< 0.001
FT4, pmol/l	−8.263	2.474	0.001

FT3: Free triiodothyronine, FT4: Free tetraiodothyronine, TSH: Thyroid-stimulating hormone

## 4. Discussion

It is evident from the present findings that BMI correlates with FT3 differently compared to FT4. Pregnant women with higher BMI are likely to have higher levels of FT3, but lower FT4. TSH levels were comparable in pregnant women with different BMI groups suggesting that BMI has no apparent influence on TSH release. These findings are supported by several previous reports [1,2,13,14,34], nonetheless contradictory results were demonstrated in other studies [15,16,19].

The pattern of changes in thyroid hormones profile in our sample is exactly similar to those demonstrated by Kahr *et al*. in 205 pregnant women at the time of delivery [1]. Kahr *et al*. results showed increased FT3, decreased FT4, and unchanged TSH levels with increasing maternal obesity. Furthermore, maternal FT3/FT4 ratio increased with higher BMI compared with normal weight cohorts. The present results and Kahr *et al*. findings are further supported by another study evaluating FT4 and TSH at 12, 24, and 36 weeks of gestation in 1035 Dutch Caucasian women [2]. At 12 weeks of gestation, BMI of Dutch Caucasian women correlated negatively with FT4, but not with TSH. According to the same study, women who gained much weight during pregnancy had lower median FT4 compared with those with normal weight gain. A comparable study among Thai women with an average 11 gestational weeks demonstrated BMI as a negative predictor of FT4 in a multiple regression model [34]. According to another report, 48% of women at the lowest FT4 decile and 22% of women at the highest FT4 decile were overweight or obese. Likewise, median FT4 is the lowermost in women with higher BMI regardless of the TSH concentration [13]. In comparison with normal and underweight groups, the obese and overweight groups investigated by Han *et al*. showed a leftward shift of FT4 distribution curves, which suggested a lower FT4 level in the groups with higher BMI [14].

Although a lack of significant association between TSH and BMI revealed by our results were in concert with several previous studies on euthyroid non-pregnant subjects [17], it disagreed with other reports [15,16,19]. In a cohort study conducted among euthyroid subjects who attended the Thyroid Clinic at the Queen Elizabeth Hospital, Birmingham, the UK, between 1987 and 2004, Manji *et al*. showed no significant relationship between BMI and serum TSH concentration [17]. In contrast, Han *et al*. confirmed a significantly higher TSH in the obese compared with the overweight group and in the overweight compared with the normal group [14]. A positive association between TSH and BMI was also demonstrated in UK pregnant women during the 7^th^-16^th^ [16] and 11^th^-13^th^ weeks of gestation [15]. A similar positive correlation between TSH levels and BMI was demonstrated among non-pregnant women [35,36].

Studies which demonstrated a positive correlation between TSH levels and BMI suggested higher leptin levels in obese subjects as a possible explanation [19]. This is because there are repeated evidence that leptin enhances TSH release [18,19]. The assumption that higher TSH in those with higher BMI is leptin-induced necessities a synonymous increase in FT4, which contradicts the well documented lower FT4 levels in the obese subjects [1,2]. Alternatively, increased iodothyronine 5´deiodinase (5´D) activity associated with obesity may give an explanation for thyroid profile in those with higher BMI [21]. It may be hypothesized that high leptin in obese and overweight subjects enhances TSH release, leading to significantly higher FT4. However, simultaneous increased in 5´D activity increases FT3 at the expense of FT4. Based on this hypothesis, high FT3 [20] and low FT4 [37] are expected to feedback differently on TSH. In high BMI subjects, increased FT3 concentration decreases TSH level while high leptin [18,19] and low FT4 levels [38] positively feedback on TSH [39]. These contradictory actions of high leptin, low FT4, and high FT3 on TSH may explain the loss of positive correlation between BMI and TSH in our study and other reports [14,17].

One of the limitations of the current study was using BMI in early pregnancy and weight gaining during pregnancy was not taken. These parameters (pre-pregnancy BMI and weight gain during pregnancy) are difficult to get in our setting. However, the early pregnancy BMI is still valid [40]. Leptin levels and 5´D activity were not evaluated in the present research, which constituted potential study limitations. Further research that correlates the thyroid hormones profile, 5´D activity and leptin levels to the variations in BMI in pregnant women is desirable to give further support to the present findings and implications.

## 5. Conclusions

The present findings demonstrate that BMI correlates with FT3 differently compared to FT4. Pregnant women with higher BMI are likely to have higher levels of FT3, but lower FT4. In contrast, TSH levels were comparable in different BMI groups.

### Disclosure Statement

No competing interests to declare.

### Funding Sources

None to declare.
